# Silencing lncRNA DUXAP8 inhibits lung adenocarcinoma progression by targeting miR-26b-5p

**DOI:** 10.1042/BSR20200884

**Published:** 2021-01-07

**Authors:** Yi Liu, Gongming Zhang, Hao Chen, Hua Wang

**Affiliations:** 1 Department of Pathology, The First People’s Hospital of Lianyungang, No.6 Zhenhua Dong Rd, Lianyungang 222002, Jiangsu Province, China; 2 Department of Medicine, The First People’s Hospital of Lianyungang, No.6 Zhenhua Dong Rd, Lianyungang 222002, Jiangsu Province, China

**Keywords:** Apoptosis, DUXAP8, Lung adenocarcinoma, MiR-26b-5p, Proliferation

## Abstract

Lung adenocarcinoma (LUAD), a common type of lung cancer, has become a popularly aggressive cancer. Long noncoding RNAs (lncRNAs) play a critical role in the pathogenesis of human cancers, while the function of double homeobox A pseudogene 8 (DUXAP8) in LUAD remains to be fully inquired. Therefore, our study was conducted to elucidate the DUXAP8 expression in LUAD and its mechanism on the biological features of LUAD cells. Loss-of-function experiments were performed to assess the function of DUXAP8 proliferation and apoptosis of H1975 and A549 cells. Functionally, silencing DUXAP8 inhibited proliferation and induced apoptosis of LUAD cells. Mechanistically, further correlation assay indicated a negative association between miR-26b-5p and DUXAP8 expression. Subsequently, we testified that DUXAP8 exerted its role in the progression and development of LUAD through targeting miR-26b-5p. In summary, our results elucidated that that DUXAP8 promoted tumor progression in LUAD by targeting miR-26b-5p, which provide a novel therapeutic target for diagnosis and therapy of LUAD.

## Introduction

Lung cancer is known to be a leading contributor of tumor-related deaths around the word, for which the 5-year survival rate is still ∼16.6% [1–5]. In this disease, 90% is divided into the non-small cell lung cancer (NSCLC), including lung adenocarcinoma (LUAD), lung squamous cell carcinoma and large cell lung cancer [6,7]. LUAD, a common type of lung cancer, has become a popularly aggressive cancer [8–10]. It is difficult to diagnose LUAD at an early stage; most patients are diagnosed at the advanced stage [11–14]. Although the therapeutic treatment for LUAD has progressed in recent years, the prognosis of LUAD patients is tremendously poor. Hence, there is an exigent necessity to probe novel targets and improve the understanding of mechanisms behind tumor progression for LUAD.

It has been found that cancer progression and tumorigenesis is related to genetic and epigenetic changes, including LUAD [15–21]. Long non-coding RNAs (LncRNAs) are a type of transcript, with more than 200 nucleotides in length, proved to be widely involved in various physiological and pathological processes [22–25]. Further, a growing number of studies have revealed that lncRNAs exerted their regulatory roles in the occurrence and progression of cancers [26–29]. For example, Jiao et al. found that lncRNA MALAT1 promotes tumor growth and metastasis by targeting miR-124/foxq1 in bladder transitional cell carcinoma [30]. In addition, lncHOXA10 drives liver TICs self-renewal and tumorigenesis via HOXA10 transcription activation [31]. Additionally, lncRNA TDRG1 has been reported that could promote cell proliferation, migration and invasion by targeting miR-326 to regulate MAPK1 expression in cervical cancer [32]. Therefore, exploring the mechanism of lncRNAs in human cancers is very significant.

Double homeobox A pseudogene 8 (DUXAP8) is located on chromosome 22q11 with 2268 bp in length [33,34]. Increasing evidences have demonstrated that DUXAP8 plays a regulatory role in many cancers, including NSCLC, gastric cancer etc [35]. Chen et al. have found that DUXAP8 promoted cell growth in renal carcinoma [36]. Moreover, DUXAP8 could regulate the proliferation and invasion of esophageal squamous cell cancer [37]. Previous study has proved that knockdown of DUXAP8 expression suppressed cell proliferation in glioma [38].

Growing evidences have demonstrated that lncRNAs could interact with miRNA to affect tumorigenesis. For example, lnc NTF3-5 promoted osteogenic differentiation of maxillary sinus membrane stem cells via sponging miR-93-3p [39]. Furthermore, lncRNA SNHG20 is involved in breast cancer cell proliferation, invasion and migration via miR-495 [40]. Pan et al. reported that lncRNA JPX regulates lung cancer tumorigenesis by activating Wnt/β-catenin signaling [41]. It is noteworthy that miR-26b-5p has been identified to be closely related to tumor growth and serves as the target of lncRNAs [42–44].

However, it is almost unknown whether DUXAP8 was a functional lncRNA in LUAD and the effect of DUXAP8 on LUAD and its underlying mechanism remains unclear. Therefore, focus of the present study is to unravel the functional mechanism of DUXAP8 in LUAD progression. First, the present study showed that the expression level of DUXAP8 was remarkably increased in LUAD tissues compared with that in adjacent tissues. Functionally, we determined functional analysis that indicated that lncRNA DUXAP8 facilitates cell proliferation and inhibited apoptosis by targeting miR-26b-5p in LUAD. Our study provides a potentially useful target for LUAD therapy.

## Materials and methods

### Tissue samples

A total of 45 tumor tissues and adjacent normal tissues were collected between June 2018 and March 2019 at the First People’s Hospital of Lianyungang. None of the patients received chemotherapy before sampling. The samples were stored in liquid nitrogen at −80°C for following experiments. The research was approved by the Ethics Committee of the First People’s Hospital of Lianyungang and informed consent was obtained from the patients. The clinical information of patients is shown in Table 1.

**Table 1:** Association of DUXAP8 expression with clinicopathological features of patients with LUAD

Characteristics	Number	Low (*n*=22)	High (*n*=23)	*P*-value
Sex				0.38
Male	29	9	20	
Female	16	6	10	
Age				0.35
≤60	28	11	17	
>60	17	5	12	
Tumor size, cm				0.026
≤4	35	17	18	
>4	10	3	7	
Pathological staging				0.016
I + II	31	15	16	
III + IV	14	6	8	
Metastasis				0.039
Yes	28	13	15	
No	17	8	9	

### Cell culture and cell transfection

Human LUAD cell lines (A549, H1299, H1975) and normal epithelial cell line (16HBE) were purchased from American Type Culture Collection (ATCC, Manassas, VA, U.S.A.). The cell lines were cultured in DMEM complemented with 10% FBS at 37°C under a moist atmosphere of 5% CO_2_. Cells were collected after 48 h for further analysis. Sh-DUXAP8 and sh-NC were obtained from GenePharma (Shanghai, China). Transfection was performed by using Lipofectamine 2000 (Invitrogen, Shanghai, China) according to the manufacturer’s instructions.

### Real-time PCR

Total RNA was isolated from tissue samples and cells with TRIzol reagent (Invitrogen), cDNA was synthesized by TaqMan Reverse Transcription Kit (Applied Biosystems). Real-time PCR (RT-PCR) was implemented on Applied Biosystems 7500 Real-Time PCR system (Applied Biosystems, Foster City, U.S.A.) utilizing SYBR Premix ExTaq™ (Life Technologies). Relative gene expression levels were calculated by the 2^−ΔΔ*C*_T_^ method and U6 were employed as internal controls for normalization.

### Western blot

Total protein was extracted from cells with RIPA buffer (Beyotime Biotechnology, Beijing, China) and the concentration of total protein was determined using the BCA Protein Assay Kit (Thermo Fisher Scientific). Proteins were separated by SDS/PAGE and transferred to PVDF membranes. Following blocked with 5% skim milk in TBST at room temperature for 1 h, probed with primary antibodies and then incubated with HRP-conjugated secondary antibodies at room temperature for 2 h. The enhanced chemiluminescence detection kit (Thermo Fisher Scientific) was used to visualize the blots. GAPDH worked as the inherent control and immunoreactive bands were quantified using ImageJ.

### Cell proliferation assay

Cell-counting kit-8 (CCK-8) and colony formation analysis were carried out to determine the effects of DUXAP8 on the proliferation of H1975 and A549 cells. In briefly, H1975 and A549 cells were seeded into 96-well plates at the density of 1 × 10^5^ per well and incubated at 37°C for 0, 24, 48 or 72 h. Then, each well was treated with 10 μl of CCK-8 and maintained at 37°C for 2 h. The optical density (OD) values were tested at a wavelength of 450 nm by the microplate reader (Bio-Tek, Winooski, U.S.A.). For colony formation assay, H1975 and A549 cells were seeded in six-well plates and grown in DMEM containing 10% FBS. The medium was replaced every 3 days. Two weeks later, the medium was discarded and then cells were fixed in 4% paraformaldehyde, stained with 0.5% Crystal Violet. Colonies were counted and photographed with a light microscope (Olympus, Tokyo, Japan).

### Flow cytometry

Cell apoptosis was determined by Annexin V-FITC kit (Beyotime Biotechnology, Shanghai, China). Cells were harvested with trypsin, washed with PBS and treated with 5 µl of annexin V-FITC and 5 µl of PI in the dark for 15 min at room temperature in the dark. Finally, cells were analyzed with an FACScan flow cytometer (BD Biosciences, Detroit, MI, U.S.A.).

### Luciferase reporter assay

Both H1975 and A549 cells were transfected with either DUXAP8 wild-type (WT) or mutated-type (Mut) promoter reporters in combination with miR-26b-5p mimic. After 48-h transfection, luciferase activity was detected by dual-luciferase reporter assay system (Promega) and luciferase intensity normalized to *Renilla* luciferase activity.

### RNA pull-down assay

Pierce™ Magnetic RNA-Protein Pull-Down Kit was used for the RNA pull-down assays. Briefly, the DUXAP8-WT, DUXAP8-Mut and NC were biotin labeled into Biotin DUXAP8-WT, DUXAP8-Mut and Biotin NC, severally. Next, cells were lysates and cultured with the biotinylated probe and M-280 streptavidin magnetic beads (Sigma–Aldrich). At last, RT-qPCR assay was used for assessing the expression of miR-26b-5p.

### Statistical analysis

Statistical analyses were performed by GraphPad Prism 5.0 and data were presented as mean ± standard deviation (SD). The differences between groups were calculated by one-way ANOVA followed by Tukey’s poc host analysis. At least three independent experiments were performed and *P*<0.05 was indicated as statistically significant.

## Results

### DUXAP8 is up-regulated in LUAD tissues and cell lines

**Figure 1:**
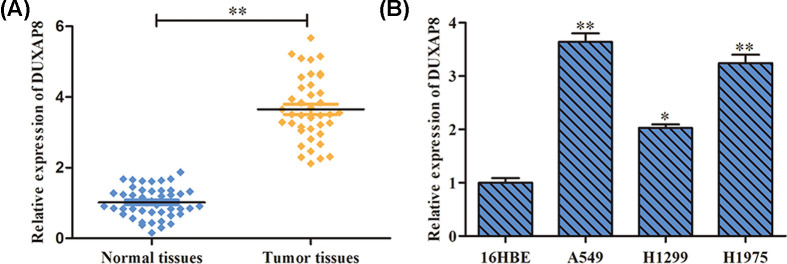
DUXAP8 is up-regulated in LUAD tissues and cell lines (**A**) The expression levels of DUXAP8 in tissue samples. **P<0.01 vs. normal tissues. (**B**) The expression levels of DUXAP8 in LUAD cell lines and normal epithelial cells. *P<0.05, **P<0.01 *vs*. MCF-10A cells. **P<0.01 *vs*. NC mimic group.

To explore the expression levels of DUXAP8 in LUAD, RT-PCR was performed to assess the expression levels of DUXAP8 in tissues and cell lines. We identified that DUXAP8 was remarkably increased in cancer samples compared with their corresponding normal samples (Figure 1A). In addition, compared with 16HBE cells, DUXAP8 expression levels was significantly up-regulated in cancer cells (A549, H1299, H1975) (Figure 1B). Based on these results, we inferred that DUXAP8 might serve as an oncogene in LUAD.

### Silencing of DUXAP8 inhibited LUAD cell proliferation

**Figure 2:**
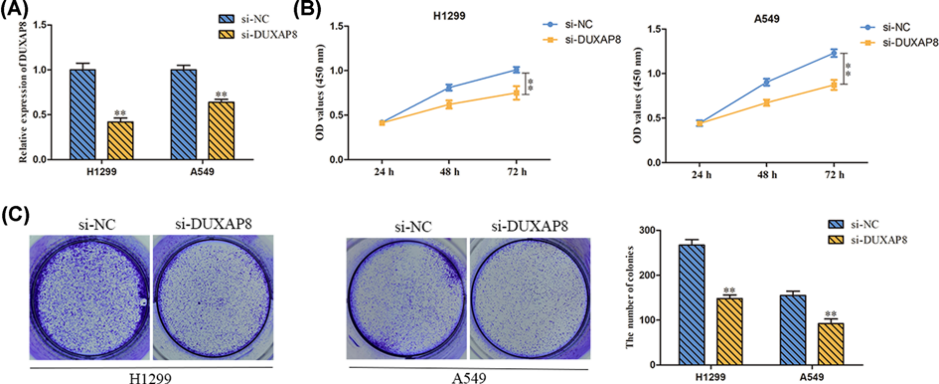
Silencing of DUXAP8 inhibited LUAD cell proliferation (**A**) Transfection efficiency of sh-NC and si-DUXAP8 in H1299 and A549 cells was detected by RT-PCR. (**B**) The influence of DUXAP8 on cell viability of H1299 and A549 cells was detected by CCK-8. (**C**) The influence of DUXAP8 on cell proliferation of H1299 and A549 cells was detected by colony formation assay. **P<0.01 *vs*. si-NC.

In order to identify the effect of DUXAP8 in LUAD, we transfected indicated cells with sh-NC, si-DUXAP8 and the efficiency of DUXAP8 expression was certified by RT-PCR. We observed that si-DUXAP8 transfection significantly decreased the mRNA levels of DUXAP8 in H1975 and A549 cells (Figure 2A). Next, we investigated the influence of DUXAP8 on cell proliferation using CCK-8 and colony formation assays. Our observations showed that silenced DUXAP8 expression caused the diminution of H1975 and A549 cell viability (Figure 2B). Consistently, the colony formation assay indicated that si-DUXAP8 restricted cell proliferation in H1975 and A549 cells (Figure 2C). Collectively, down-regulation expression of DUXAP8 could inhibit cell proliferation in LUAD.

### Silencing of DUXAP8 promoted cell apoptosis

**Figure 3:**
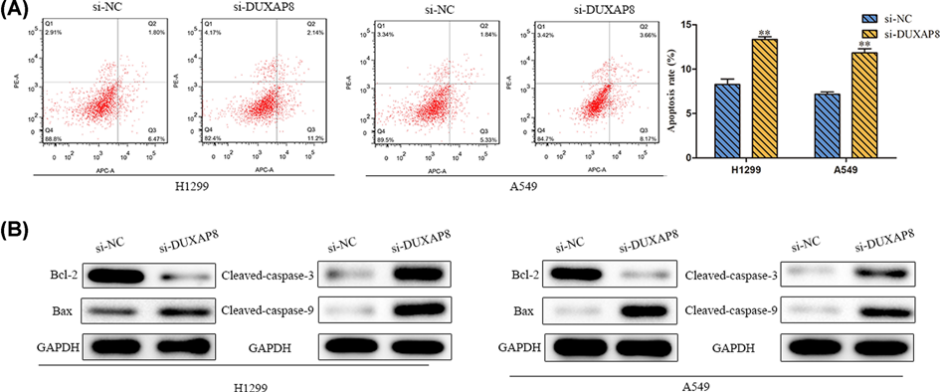
Silencing of DUXAP8 promoted cell apoptosis (**A**) Cell apoptosis was detected by flow cytometry. (**B**) Western blot assay was used to detect the expression levels of Bax, Bcl-2, Cleaved-caspase-3 and Cleaved-caspase-9. GAPDH was used as the normalized control. **P<0.01 *vs*. si-NC.

Flow cytometric analyses elucidated that the percentage of apoptotic cells was enhanced by si-DUXAP8 compared with si-NC group (Figure 3A). Consistently, the expression levels of pro-apoptotic proteins (Bax, Cleaved-caspase-3 and Cleaved-caspase-9) were all increased, whereas Bcl-2, the anti-apoptotic gene was memorably down-regulated in si-DUXAP8 group (Figure 3B). Collectively, down-regulation of DUXAP8 expression could facilitate cell apoptosis in LUAD.

### MiR-26b-5p was a downstream target of DUXAP8

**Figure 4:**
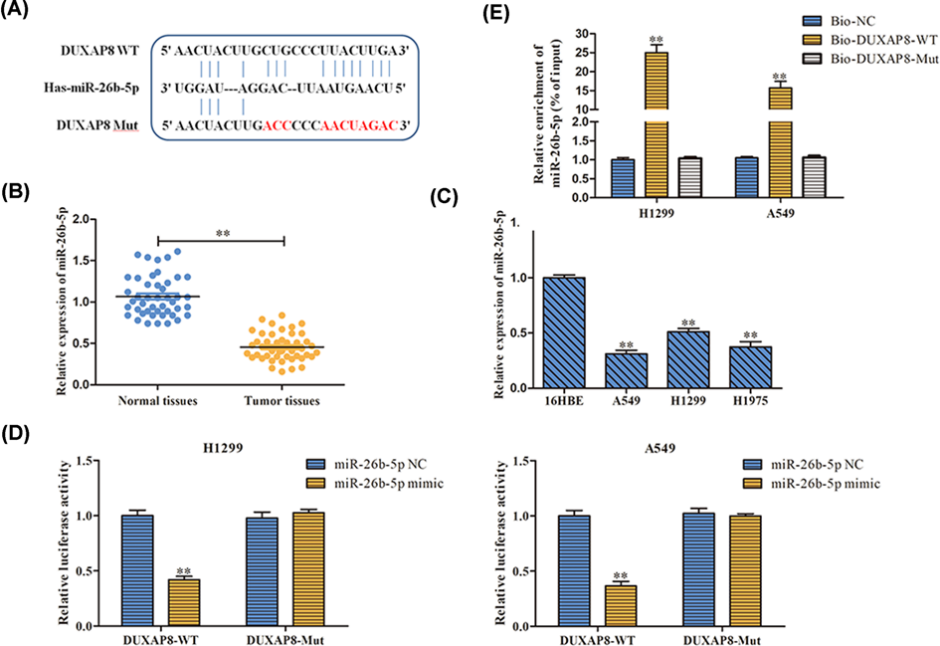
MiR-26b-5p was a downstream target of DUXAP8 (**A**) The target relationship between DUXAP8 and miR-26b-5p was predicted by TargetScan website. (**B,C**) The expression of miR-26b-5p in LUAD tissues and cells was detected by RT-PCR. (**D**) The targeting relationship between DUXAP8 and miR-26b-5p was verified through dual luciferase reporter gene assay. (**E**) RNA pull-down assay was utilized to verify the relationship between DUXAP8 and miR-26b-5p. **P<0.01 *vs*. si-NC.

MiR-26b-5p was predicted as the putative target of DUXAP8 according to bioinformatics analysis (Figure 4A). We first verified the level of miR-26b-5p in tissues and cell lines. In contrast with DUXAP8 expression, miR-26b-5p expression level was dramatically decreased in LUAD tissues and cells (Figure 4B,C). As a result, we intended to explore the association between DUXAP8 and miR-26b-5p. The luciferase reporter assay showed that miR-26b-5p mimic repressed the relative luciferase activities containing the DUXAP8-WT, while no obvious alteration was viewed in the mutant form of DUXAP8 (Figure 4D). Moreover, pull-down assay was conducted and result showed that miR-26b-5p expression was more enriched by biotinylated DUXAP8-WT than DUXAP8-Mut or NC groups (Figure 4E). These data revealed that DUXAP8 negatively regulated miR-26b-5p expression by directly targeting the 3′-UTR of miR-26b-5p.

### DUXAP8 facilitated cell progression via targeting miR-26b-5p

**Figure 5:**
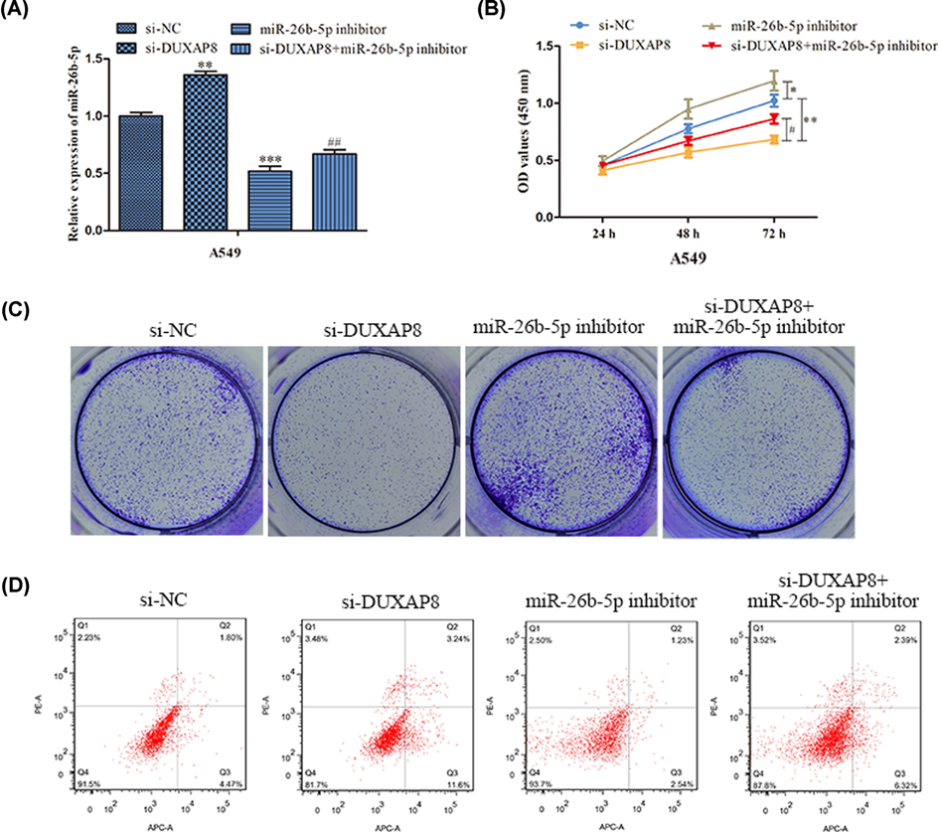
The role of miR-26b-5p in LUAD carcinogenesis was mediated by DUXAP8 (**A**) The expression levels of miR-26b-5p were measured in H1975 and A549 cells. (**B**) Cell viability was measured by CCK-8. (**C**) EdU assay was performed to assess the proliferation of H1975 and A549 cells. (**D**) Cell apoptosis were explored via flow cytometry. All results were presented as mean ± SD from at least three independent assays. *P<0.05, **P<0.01, ***P<0.001 *vs*. si-NC. ^#^*P*<0.05, ^##^*P*<0.01 *vs*. si-DUXAP8.

Based on the above findings, we performed the rescue assays to certify whether DUXAP8 exerted its oncogenic function by modulation of miR-26b-5p. MiR-26b-5p expression levels were observably elevated in H1975 and A549 cells after transfected with si-NC, si-DUXAP8, miR-26b-5p inhibitor or si-DUXAP8+miR-26b-5p inhibitor (Figure 5A). CCK-8 assay illustrated that cell viability was depressed by down-regulation of DUXAP8 expression and subsequently recovered by miR-26b-5p inhibitor (Figure 5B). Concordant with CCK-8 assay, colony formation assay demonstrated that inhibition of miR-26b-5p expression abrogated the anti-proliferative effects of si-DUXAP8 (Figure 5C). As expected, the anti-apoptotic functions of DUXAP8 in H1975 and A549 cells were partially reversed by miR-26b-5p (Figure 5D). To this end, these results provided strong evidence that DUXAP8 functioned as a tumor promoter in LUAD progression via suppression of miR-26b-5p.

## Discussion

Lung cancer is a malignant cancer in the world with high morbidity and high mortality. LUAD is a common subtype of lung cancer with stagnant improvement in prognosis during past decades despite the treatment progress [45]. Despite some progress in related treatments in recent years, the overall 5-year survival rate of advanced lung cancer is still less than 15%. Accordingly, elucidating the underlying mechanism of LUAD to discover effective diagnostic and prognostic biomarkers is conducive to the improvement of LUAD therapy.

Over the past decades, mounting studies have reported that lncRNAs play important roles in human cancers progression, including LUAD. Peng et al. showed that lncRNA CRNDE promotes colorectal cancer cell proliferation and chemoresistance via miR-181a-5p-mediated regulation of Wnt/β-catenin signaling [46]. In addition, lncRNA NORAD has been reported that contributes to colorectal cancer progression by inhibition of miR-202-5p [47]. Moreover, lncRNA XIST promotes human LUAD cells to cisplatin resistance via let-7i/BAG-1 axis [48].

Nevertheless, there are still numerous lncRNAs that need to be elucidated. In the present study, we focused on the biological function of DUXAP8 in the development of LUAD. In the current study, we first prospected the expression of DUXAP8 in LUAD tissues and cells. In contrast with normal tissues and cells, DUXAP8 was highly expressed in LUAD cells. These data were consistent with previous findings showing DUXAP8 exerted its effect as a tumor promoter in regulating cancer progress [49,50]. Thereafter, DUXAP8 was silenced in H1975 and A549 cells to carry out loss-of-function experiments. Our results expounded that depletion of DUXAP8 suppressed cell proliferation and promoted apoptosis. However, the mechanism about how DUXAP8 is involved in progression of LUAD remains unclear.

Accumulating researches suggested that lncRNAs regulated cell functions through interacting with miRNA [51]. Furthermore, growing studies emphasize that miRNAs are regarded as core mediators in progression and development of multiple malignant tumors via functioning as oncogenes or tumor suppressors [52,53]. By utilization of bioinformatics tool miR-26b-5p was found to own binding sites with DUXAP8. In addition, we carried out luciferase reporter assay to identify the correlation between miR-26b-5p and DUXAP8 in LUAD cells, discovered that miR-26b-5p was negatively regulated by DUXAP8. Moreover, rescue experiments unveiled that depletion of miR-26b-5p blocked the inhibitory effects of miR-26b-5p down-expression on cell proliferation and apoptosis of LUAD.

In summary, we unraveled that silencing DUXAP8 expression suppresses cell proliferation and enhanced apoptosis by targeting miR-26b-5p, which serves as a cancer facilitator in LUAD. To the best of our knowledge, this is the first investigation to shed light on the potential and molecular mechanism of DUXAP8 in LUAD. Our findings represent a potential therapeutic target for the treatment of LUAD, whether it involves more complicated regulation is still to be explored by us and other researchers. We will further make deeper and more detailed studies about regulation mechanism of DUXAP8 on LUAD in the future work.

## Data Availability

The datasets used and/or analyzed for the current study are available from the corresponding author upon reasonable request.

## Abbreviations

Bax: BCL2 associated X, apoptosis regulator
Bcl-2: BCL2 apoptosis regulator
CCK-8: cell-counting kit-8
CRNDE: colorectal neoplasia differentially expressed
DUXAP8: double homeobox A pseudogene 8
foxq1: forkhead box Q1
GAPDH: glyceraldehyde-3-phosphate dehydrogenase
HOXA10: homeobox A10
HRP: horseradish peroxidase
JPX: JPX transcript
lncRNA: long non-coding RNA
LUAD: lung adenocarcinoma
MALAT1: metastasis associated lung adenocarcinoma transcript 1
NORAD: non-coding RNA activated by DNA damage
NSCLC: non-small cell lung cancer
PI: propidium iodide
RT-qPCR: real-time quantitative PCR
SNHG20: small nucleolar RNA host gene 20
TBST: Tris Buffered saline Tween
TDRG1: testis development related 1
TIC: tumor initiating cells
XIST: X inactive specific transcript
